# Mr. Dawson, on Dr. Bellamy's Case of Diseased Bladder

**Published:** 1806-09

**Authors:** G. P. Dawson

**Affiliations:** Sunderland


### *To the Editors of the Medical and Physical Journal.*

Gentlemen,

TO expose error, to notice inconsistency, or to animadvert on what is imprudent, is an ungrateful task, and one which a candid and generous heart will always undertake with regret. It seems to indicate an asperity which is illiberal, a minuteness of investigation which is severe, and to display a triumph on the detection of imperfection not compatible with liberality. But these considerations should not prevent us from giving birth to the dictates of our judgment, or to the promulgation of that which may tend to the exposition of error, or to the elucidation of misconception. Dr. Bellamy, in relating his case of diseased bladder, has invited criticism by the strangeness of his language, the singularity of his practice, the originality of his reflections, and the ambiguity of his descriptions. This he has done so palpably as to render it irksome. I imagine him to have pursued a practice, I conceive to be improper, and to have hazarded remarks I believe to be erroneous.

My design is to comment on the former, and to refute the latter. To some it may appear invidious and unnecessary, but an enlightened mind will view it with candour and complacency. It is an attempt to counteract what may may be injurious to the indolent or the ingnorant, who rely implicitly on whatever appears in print. Dr. Bellamy says, " Mr. Andrew Sharp, aged about thirty-eight, came under my care on the 22d of January 1806, for an affection in perinæo; a considerable hardness to be felt, and some enlargement to be seen at the root of the penis, or rather at the bulb of the urethra; *feels much pain when touched, and a good deal of internal distress, with sense of fulness and aching, even when the parts are at rest*; *a great deal more when he evacuates urine or stools; the former, runs off in a small narrow stream; does not stop, but requires a little time to begin.* An old complaint; was under my care about six months ago, then relieved by small mercurial frictions on the part; is *the effect believed of an old gonorrhœa some years ago, and by quacking with improper injections*; occasionally a little gleet; looks ill with it; there is no redness, or heat, *or sign of inflammation*; but supposed to be a *chronic thickening or enlargement*: probably the prostrate gland is diseased. Utatr. ung. hydrag. gr. v. bis in die part. affect. heri haust. cath. fotus com. et rep. p. r. n." It is obvious he had stricture, which being disregarded had occasioned these appearances and symptoms. Tumefaction in the perinæum and consequent fistula is a common consequence of neglected stricture.

Dr. Bellamy observes, there is no *sign of inflammation*." Was there not every sign? Was not painful " *when touched, a good deal of internal distress, with a sense of fulness and aching when the parts are at rest*," a demonstrable proof of inflammation? Could there be a better? Did not the event prove the fact? The Doctor supposes the swelling " to be *a chronic thickening or enlargement*." Why *chronic*? Are *chronic* enlargements designated by " *much pain when touched, internal distress, and a sense of fulness and aching*." Certainly not. Why mercurial friction was employed I cannot conceive. The perinæal affection was not venereal, why therefore have recourse to mercury? Admitting, for the sake of argument, though it exceeds credence, that mercury was indicated, of what use could *five grains* of mercurial ointment be rubbed twice a day upon a part *painful and inflamed*? The question would puzzle the Priestess of the Delphic God, were she skilled in surgery. The friction was alone sufficient to increase the inflammation, independent of the mercury, a medicine highly irritating. Its injurious effect is shewn on the 23d, when the " Swelling is undoubtedly larger, yet not hot or red, but more extensive, and very apparent, with a great a great sense of fulness; pain in making water, but not interrupted, except being in a small stream, as is always the case with him, more or less; the physic acted well. ℞. Fotus, et ung. hydrag." The frictions are continued; and on the 24th "notwithstanding my attempt to promote *absorption,* the tumour increases very much, *and even with a degree of inflammation; great pain;* looks, and feels weak, pale, &c.;" still shall persevere to *repel,* for fear of fistula. ℞. *Ung. hydrag. fot. &c."* Dr. Bellamy now admits there is inflammation.

By fomentations, I understand a warm application; and it is for me yet to learn, that warmth and mercurial ointment were probable means of repelling the inflammation, evidently the indication of cure. The Doctor speaks of absorption. To me this seems an ambiguous term. What wished he to absorb? The termination of the case proved the swelling to be no *chronic enlargement.* Indeed, how could it be a *chronic* one? Neglected stricture in the urethra had produced inflammation and tumefaction in the perinæum, and the object was to overcome the stricture (the exciting cause) by the cautious and repeated introduction of different sized bougies, and to remove the inflammation and swelling by cold water, leeches, and a strict observance of the antiphlogistic regimen. This would seem the only rational method of treatment, and to hold out the fairest prospect of success. But though the patient is pale and *weak,* the tumour *large,* with *inflammation* and *great pain,* Dr. Bellamy will still persevere to *repel* it, and use mercurial frictions. Let me be excused for recurring to a pratice so pertinaciously pursued. To repel inflammation the Doctor employs mercurial ointment. To *disperse* a tumour, *painful* and *inflamed,* he rubs upon *its surface five grains twice a day.* He does this to prevent suppuration and a fistula in perinæo. Could he have devised a method more likely to produce them? When blue ointment is rubbed on the thigh for a veneral purpose, does it not render the part red and uneasy, so much so as to induce the person to select another place for its application? Could it fail to have a similar effect in a swelling attended with apparent inflammation and great pain?

When did mercury ever subdue inflammation? It will cure a venereal action, but it was never known to lessen an inflammatory one. On the 25th, the "Tumour is still large, very painful all night, and *extremely sensible to the touch;* feels most relief by fomentation. *I rather hope* it

*it will break internally, and thus save an external wound or fistula in perinæo; yet even this may cause destruction and find its way through; but on the whole, better so than fistula in perinæo.* ℞. *Ungt.* fotus, &c."

These lines are pregnant with inconsistency and error. I must be permitted to dwell on, and examine them minutely, lest they should be disregarded and their fallacy remain unexposed. Dr. Bellamy hopes the abscess will "*break internally, and save an external wound or fistula in perinæo.*" Can the reader refrain from smiling? How could the abscess break *internally*? Into the *urethra* or into the *bladder*? Impossible! What was to become of the contained fluid? If the abscess could break *internally*, the matter must find its way into the *urethra* and the *bladder* by *ulceration*. Could vitality sustain an injury so deadly? How was the matter to be evacuated *without an external wound*? Was it to be drawn off by a catheter? Or was the bladder to be tapped? Yet that could not be intended, as the operation would require an *external wound*. I can give no satisfactory answer to my queries, and must refer to Dr. Bellamy for their solution. Pardon me, Gentlemen, for discussing what is absolutely impossible. The abscess could not break without an external opening. It was to be looked for; it was the only method of cure. The reverse, admitting the possibility, would have been mortal. The Doctor says, if the abscess does *break internally, it "may cause destruction, and find its way through; but on the whole, better so than fistula in perinæo."* This observation must excite the laughter of every man, whose laughter is not suppressed by delicacy.

Dr. Bellamy seriously tells us, it would be better for a perinæal abscess to break internally, cause destruction, and ulcerate through parts essential to life, than to be opened in the perinæum, a part perfectly safe, and where the matter would have free egress, without inconvenience or danger. The idea of a fistula in perinæo appears to have been strangely terrific to his imagination. But surely, without reason; it is an unpleasant, but certainly not a remarkably serious disease. The mercurial frictions are this day employed. Dr. Bellamy used them to *repel* a swelling in perinæo. He fails in the attempt; and although he admits this swelling will *suppurate*, yet he continues their application. Can any thing be more inconsistent, or more pernicious in its tendency? In vain shall we seek a precedent for a practice that rubs mercurial ointment on a suppurating and a painful tumour.

On

On the 26th, the "abscess is very large, nearly as large as a fist, extending quite to the anus, and on its sides, a little thence up the perinæum, about two inches; *will probably contain more than a pint of matter; begins to fluctuate, feels soft and pointing.*" Now was the time at which a large opening should have been made by a lancet. But the Doctor, instead of doing this gravely, remarks, "Keep parts very clean, and him rather low; now must *promote suppuration*; *no hopes of absorption*. Appr. cataplasm a emoll. ter die cum fot. com. &c." Why "*promote suppuration?* Had not suppuration taken place? Certainly, Dr. Bellamy has said the tumour "*fluctuates, feels soft and pointing.*" Why, therefore, employ warm poultices to promote a process already completed? After suppuration they are prejudicial by relaxing the parts. On the *very day* the Doctor had relinquished all "*hopes of absorption,*" and attempted to "*promote suppuration,*" "the abscess broke," and by *ulceration* produced an opening large enough to "*admit the little finger.*" Does not this shew the eminent advantage that would have attended a free incision by the lancet?

Dr. Bellamy, in his subsequent description of the state of the parts, is so obscure, as to render himself unintelligible; and this compels me to comment on his daily reports much less minutely than I intended. After the abscess broke, a catheter was passed, "*tolerably easy,*" into the bladder. Why a catheter? Would not a bougie have been more appropriate, and less likely to occasion injury? Is it not singular, that no catheter or bougie was introduced previous to the swelling suppurating

On the 28th, it is remarked, "all I can do to prevent, is to keep the parts very clean, support the system, and watch the general health or *action of any virus.*" What *virus?* No venereal virus existed. What is meant by the "*action of any virus?* The catheter was again passed "but with much *more difficulty*; blood came through it from *evident obstructions at the curve of the urethra*; after *several attempts*, and *some force*, got into the bladder." These lines do no credit to the operator's prudence. It is never advisable to employ force in the introduction of a catheter. On the state of the abscess, where Dr. Bellamy speaks of "*a blackish brown eschar*" breaking through, and "*forming a second hole,*" and of its parietes "*being disorganized as if acted on by caustic,*" together with a sinus near the anus, I shall forbear comment; and simply suggest the possibility of all this being prevented by the relin-

 quishment quishment of the mercurial frictions, and by a large opening with a lancet. The Doctor hopes "the destructive action of the abscess, though at first *excited by venereal cause,* and obstruction in the *urethra,* will not extend to destroy *its coats.*" How was the abscess "*excited by a venereal cause?*" By venereal, I understand syphilis. It will not be affirmed stricture or gonorrhœa are the consequence of lues venerea, or were ever considered as symptoms. How then can this gentleman, without an obvious dereliction of propriety, assert the abscess was *excited by a venereal cause?* He observes, the abscess at first, excited by "obstruction in the *urethra,* will not extend to destroy *its coats:* in fact, towards *it* the probe scarce passes." On reading thus far, it is evident that by "*its coats*" is meant those of the *urethra.* It cannot be otherwise. Now the urethra has *no* coats; it is one continued, smooth, vascular membrane. Dr. Bellamy says, it is "too soon yet to prognosticate or look to radical means; (could we look to radical means too early, when the state of the parts is considered?) continue still to cleanse, palliate, and reduce the active state and effects of inflammation by poultices, &c." "He is weak, reduced, and pale; begin the bark."

On the 29th, after much ambiguity of description, and originality of reflection, the Doctor remarks, it is in his patient's favour to have his mouth affected by the mercurial frictions; hence it is clear he believed the disease to have originated from lues venerea, and cannot accuse me of misrepresentation. How a man, pale, and weak by a large abscess, with sinus, and obstruction in the urethra, indisputably not produced by a venereal taint, could be benefited by a process not necessary, and unquestionably debilitating and distressing, far exceeds my comprehension.

Previous to the 30th, Dr. Bellamy has said, no urine passed by the abscess; but on this day, he informs us, about half an ounce was evacuated. He consoles himself, by observing, it was "a reasonable event to expect, there evidently existing narrowness of the canal, passing the catheter requiring *considerable* trouble. *The obstructions may be felt breaking down under it.*" Was not an opening made through the urethra into the abscess by the point of the catheter? Illiberality or invidiousness I disclaim, but truth demands the question. If I wrong Dr. Bellamy by the supposition, I am sorry for it; but if he wished to have prevented it, he should have been more guarded in his language.

language. He first speaks of the catheter passing "*tolerably easy into the bladder;*" next, with "*several attempts, and some force;*" and lastly, of its introduction "*requiring considerable trouble, and that the obstructions may be felt breaking down under it.*" The candid and enlightened reader will determine. To criticise further would be useless: two days afterwards the parts became gangrenous, and death would undoubtedly close the scene. I wait not to hear Dr. Bellamy's conclusion of this unfortunate case; my object was not to comment on its nature, but to notice his treatment and observations. Whether the bladder was diseased or not, is of little consequence. The exciting cause was doubtless stricture; and neglected stricture will occasion disease in the bladder. The Doctor cannot be offended at what I have written, or think I have taken any unfair advantage of his statement. His case was published "for criticism," and to criticism it has been subjected. I presume not to say his practice occasioned the melancholy issue of the case; probably the same might have occurred under any treatment; but I must aver, I do not believe his to have been agreeable to the rules of modern surgery. I may be mistaken, but I think Dr. Bellamy an old practitioner, and, no doubt, experienced and well informed; but he has, in this instance, appeared before the medical world under unfavourable circumstances, and committed himself to the mercy of those who, either from cynicism or utility, delight in criticism. I can assure this gentleman I have been actuated by no motive but those of candour and veracity. If the liberty I have taken excites his displeasure, it was neither my intention nor my wish; and, if I have derogated in personal respect to him, or made unbecoming comments on his paper, I ask his pardon, for it was diametrically opposite to my design.

I am, &c.

*Sunderland, August* 10, 1806. G. P. DAWSON.

